# *Ophirina amphinema* n. gen., n. sp., a New Deeply Branching Discobid with Phylogenetic Affinity to Jakobids

**DOI:** 10.1038/s41598-018-34504-6

**Published:** 2018-11-01

**Authors:** Akinori Yabuki, Yangtsho Gyaltshen, Aaron A. Heiss, Katsunori Fujikura, Eunsoo Kim

**Affiliations:** 1 Department of Marine Biodiversity Research, Japan Agency for Marine-Earth Science and Technology (JAMSTEC), Yokosuka, Japan; 2 Division of Invertebrate Zoology and Sackler Institute for Comparative Genomics, American Museum of Natural History, New York, USA

## Abstract

We report a novel nanoflagellate, *Ophirina amphinema* n. gen. n. sp., isolated from a lagoon of the Solomon Islands. The flagellate displays ‘typical excavate’ morphological characteristics, such as the presence of a ventral feeding groove with vanes on the posterior flagellum. The cell is ca. 4 µm in length, bears two flagella, and has a single mitochondrion with flat to discoid cristae. The flagellate exists in two morphotypes: a suspension-feeder, which bears flagella that are about the length of the cell, and a swimmer, which has longer flagella. In a tree based on the analysis of 156 proteins, *Ophirina* is sister to jakobids, with moderate bootstrap support. *Ophirina* has some ultrastructural (e.g. B-fibre associated with the posterior basal body) and mtDNA (e.g. *rpoA*–*D*) features in common with jakobids. Yet, other morphological features, including the crista morphology and presence of two flagellar vanes, rather connect *Ophirina* to non-jakobid or non-discobid excavates. *Ophirina amphinema* has some unique features, such as an unusual segmented core structure within the basal bodies and a rightward-oriented dorsal fan. Thus, *Ophirina* represents a new deeply-branching member of Discoba, and its mosaic morphological characteristics may illuminate aspects of the ancestral eukaryotic cellular body plan.

## Introduction

The origin of eukaryotes, and their subsequent early diversification into major extant lineages, are each fundamentally important yet challenging topics in evolutionary biological studies. Phylogenetic relationships among major groups of eukaryotes remain much debated, and a consensus concerning the position of the eukaryotic root has not yet been reached. One hypothesis, based on the results of both large-scale phylogenetic analyses and comparative cytoskeletal structure, suggests that the root lies within or near Discoba (e.g.^1,2^). Discoba, together with Metamonada and Malawimonadida, comprise the “supergroup” Excavata^3^. Discoba is itself composed of four subgroups, Euglenozoa, Heterolobosea, Tsukubamonadida and Jakobida, which are morphologically and ultrastructurally distinct from one another^3–5^. Jakobida is of particular evolutionary interest with regard to its mitochondrial genome, the most bacterium-like known amongst eukaryotes^6,7^. For example, some subunits of RNA polymerase (i.e., *rpo A–D*) are encoded in the mitochondrial genomes of jakobids, but not in other eukaryotes^8^; and Shine-Dalgarno motifs are found in the mitochondrial genomes of only a few eukaryotic lineages, including jakobids^7,9^. Nevertheless, despite their evolutionary significance, many lineages of discobid protists still remain undersampled (e.g.^10,11^), as is the case with other probably-deeply-branching eukaryotic groups, such as mantamonads and ancyromonads. Within discobids, Heterolobosea and Euglenozoa, collectively called Discicristata^12^, have each been studied for many decades, but the two other discobid lineages (Tsukubamonadida and Jakobida) have been little explored. Tsukubamonadida was only recently (2011) established, and is currently represented by only one known species, *Tsukubamonas globosa*^4^. To date, the diversity of tsukubamonads has not been investigated from environmental DNA samples. The diversity within the order Jakobida is known to be undersampled: a recent study based on environmental sequences showed that a large number of jakobids remain to be cultured^13^. However, all currently-known jakobids, cultured and uncultured, can be assigned to one of two phylogenetically distinct suborders, Histionina and Andalucina. Histionina contains all sessile lorica-forming members of the group, and Andalucina contains all anaerobic members; however, both groups also contain free-swimming aerobes^13^. Although all jakobids feature the complete set of ‘typical excavate’ morphology, the two suborders are distinguished by additional ultrastructural characteristics in cultured members^13^. Given the possibility that the discobids may be an early-diverging lineage of eukaryotes, currently-unidentified members of the group may hold key information for considering the early evolution of not only Discoba, but also of all eukaryotes. The identification of such novel discobids has therefore been a much-anticipated protistological subject.

Here we report a new free-living, aerobic discobid flagellate (strain JB) from a shallow lagoon in the Solomon Islands. Its precise phylogenetic position could not be determined by light microscopy or by 18S rRNA gene phylogeny. On the other hand, phylogenomic analyses based on a 156-protein dataset placed strain JB sister to all previously-known jakobids. Our electron-microscopic observations revealed that strain JB has a novel combination of ultrastructural characteristics, although the general architecture of its cytoskeleton is similar to that of jakobids. Based on these findings, we formally describe strain JB as *Ophirina amphinema* gen. et sp. nov., and place it within a new suborder of Jakobida. We also sequenced the complete mitochondrial genome of *O*. *amphinema* for the present study, and discuss parallel losses of mitochondrial genes within Jakobida.

## Results

### Gross morphology

**Figure 1:**
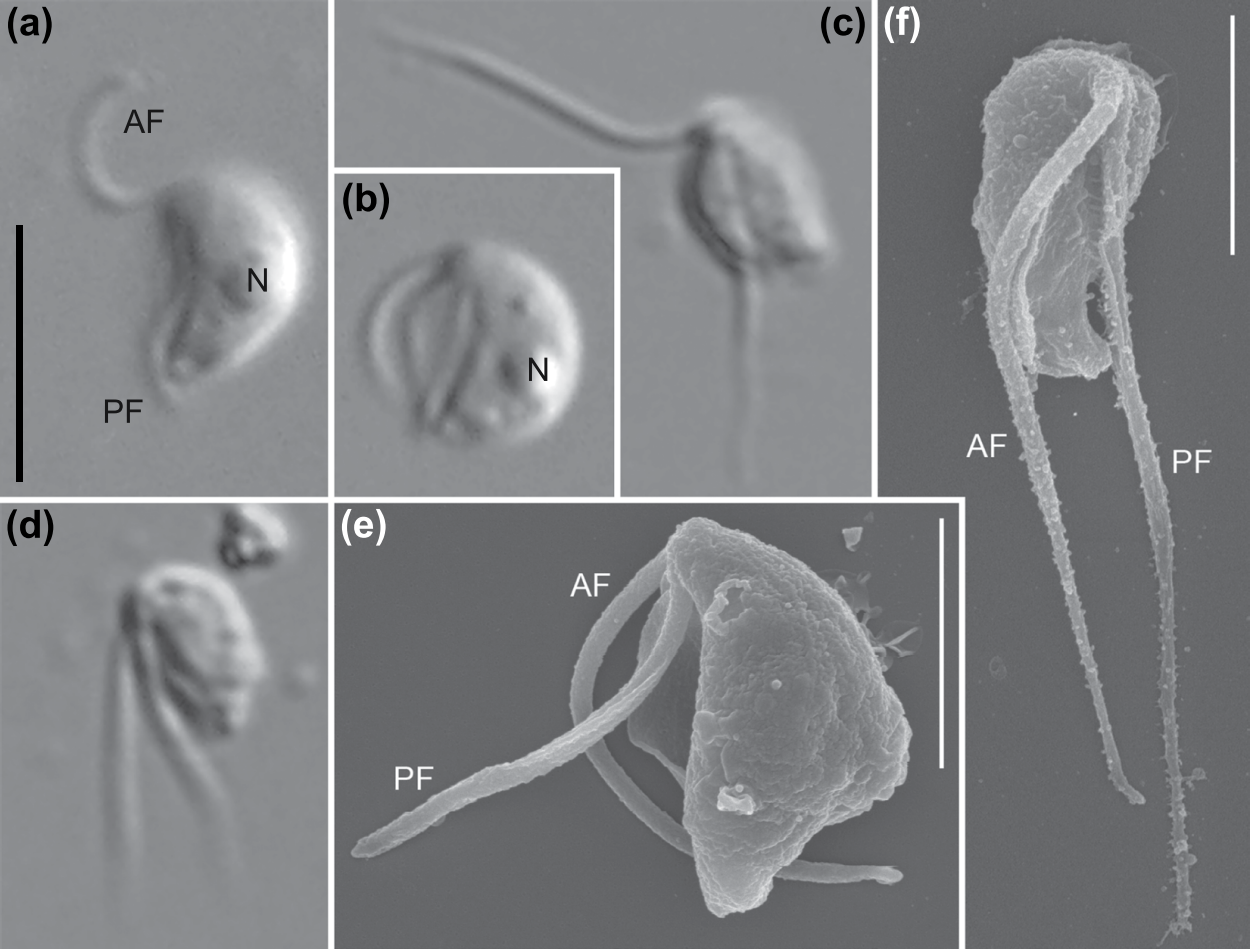
Light (**a**–**d**) and scanning electron (**e**,**f**) micrographs of *Ophirina amphinema* gen. et sp. nov. Light micrographs employed differential interference (Nomarski) optics. Scanning electron micrographs were prepared differently: (**e**) is from ‘fast’ prep, (**f**) from ‘slow’ prep (see text for details). Anterior is to top of page in all panels, and dorsal is to right of page in all panels except (**f**). (**a**,**b**,**e**) Images of suspension feeder. Both anterior and posterior flagella are equal to cell length. Nucleus (n) is visible (**a**,**b**). Flagellar vanes are visible on the posterior flagellum (**e**). (**c**,**d**,**f**) Images of swimmer. Both anterior and posterior flagella are approximately twice as long as cell length. Both flagella are inserted at anterior apex; posterior flagellum runs adjacent to ventral feeding groove. Scale bars = 5 µm (**a**), 2 µm (**e**,**f**). Scale bar in (**a**) is applicable to (**b**–**d**).

Strain JB (the abbreviation referring to its [j]umping motion and [b]ean-shaped cell) exhibited dimorphism under our growth conditions. Repeated efforts to isolate one of the two morphologies invariably resulted in cultures of both types; both were present and obvious in all cultures. One type was suspended in the water column and bore two flagella of about the cell’s length (Fig. 1a,b) While its flagella did beat, it exhibited little if any translational movement; instead it appeared to be flipping from one side to the other with a ‘jumping’ motion (Supplementary Video 1). The other was an actively swimming cell with two longer flagella, each about twice the length of the cell (Fig. 1c,d). Both cell types bore a ventral feeding groove, and always vibrated or flipped rapidly about the cell’s anteroposterior axis (Supplementary Videos 1 and 2). Besides the flagellar length, we did not notice any morphological difference between the two cell types under light microscopy. It is not presently clear when and how the flagellate transforms from one type to the other. The cells were 3.9 ± 0.4 (SD) µm in length (n = 20, 10 cells sampled per each cell type). Both flagella emerged from the anterior end (Fig. 1). We observed no other cell morphologies, such as amoeboid cells or cysts.

### Ultrastructure

**Figure 2:**
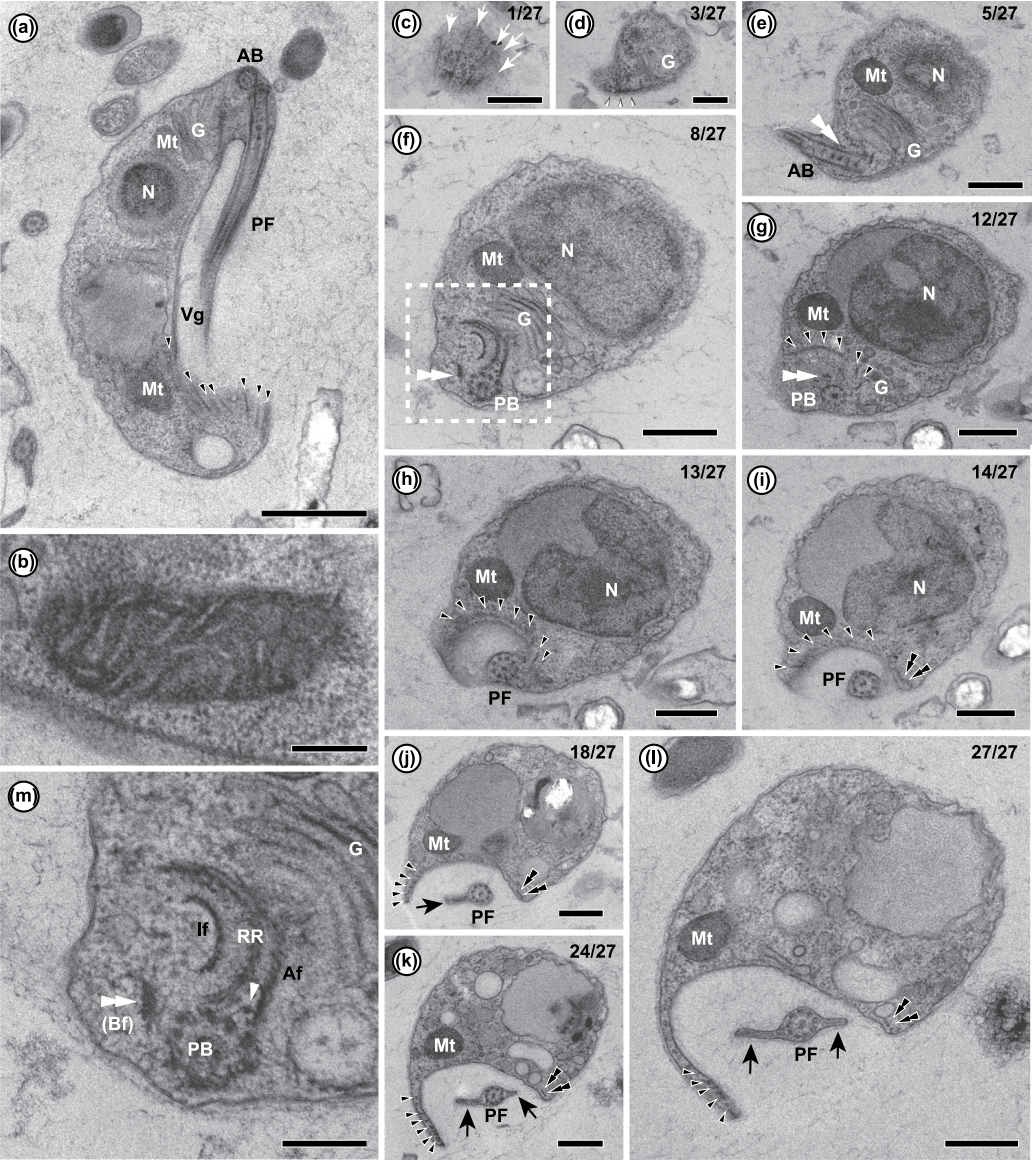
Transmission electron micrographs of *Ophirina amphinema*. AB, anterior basal body; PF, posterior flagellum; G, Golgi apparatus; Mt, mitochondrion; N, nucleus; vg, ventral feeding groove; PB, posterior basal body; Af, A-fibre; Bf, B-fibre (also indicated by double white arrowhead); If, I-fibre; RR, right root. Black and white arrows indicate flagellar vane and dorsal fan microtubules, respectively. Black and white arrowheads indicate right root microtubules and singlet root microtubule, respectively. Double arrowheads indicate left root microtubules. Small white arrowhead indicates singlet root microtubule and white narrow arrowheads with black rim indicate microtubule organising centre (MTOC). (**a**) Longitudinal section of whole cell, showing Golgi apparatus near anterior apex and microtubules supporting ventral feeding groove. This is same cell and section seen in Fig. 4c. Scale bar = 1 µm. (**b**) High magnification view of mitochondrion, showing flat-to-discoid cristae. Scale bar = 200 nm. (**c**–**i**) 10 selected micrographs from series of 27 sections (indicated by number in upper right of each panel), showing general structure of flagellar apparatus. Scale bars = 500 µm. (**m**) Enlarged view of ultrastructure around posterior basal body, corresponding to dashed box in Fig. 2j. Unilateral row of RR microtubules in “U-shape” near basal body, sandwiched by A-fibre and I-fibre. Singlet root originates amidst posterior basal body, right root and A-fibre. Scale bar = 200 nm.

The cell surface was smooth, without scales, spines, or other features (Fig. 1e,f). The single nucleus was located in the anterior to central area of the cell, and a Golgi apparatus was located between the nucleus and basal bodies (Fig. 2a,e–g). Each cell appeared to have a single mitochondrion, which was elongate and on the right side of the cell, in close association with the nucleus at its anterior end (Fig. 2e–l) and extending most of the way to the posterior of the cell (Fig. 2a). The cristae of the mitochondrion appeared flat in most sections, but in some sections, some cristae were not connected to the inner membrane, and in some cases appeared circular, suggesting instead that the cristae were discoidal (Fig. 2b).

**Figure 3:**
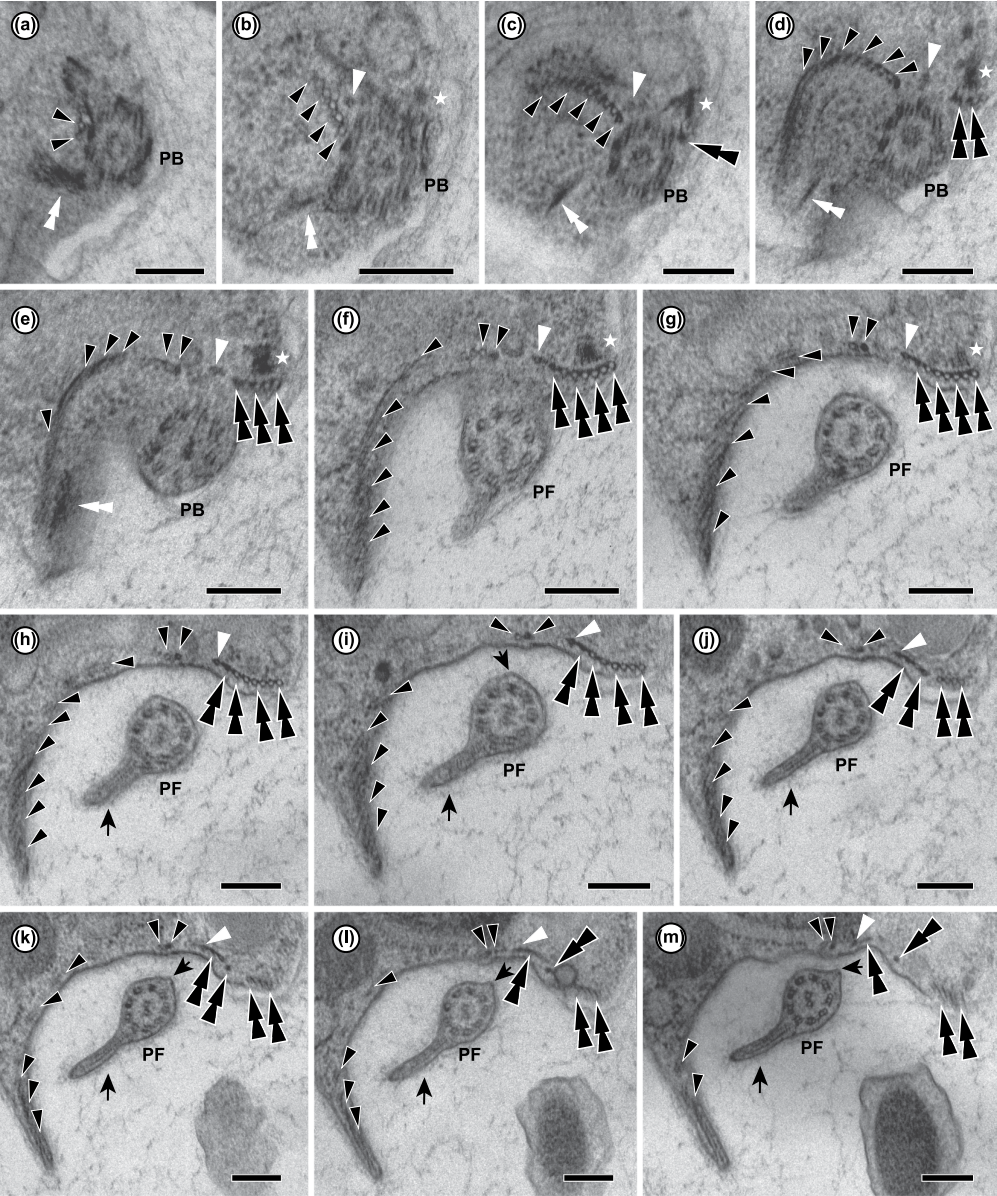
13 consecutive serial sections of *Ophirina amphinema* showing ultrastructure of flagellar apparatus. PB, posterior basal body; PF, posterior flagellum. Black and white arrowheads indicate right root microtubule and singlet root microtubule, respectively. Double black arrowheads and double white arrowheads indicate left root microtubules and B-fibre, respectively. Stars indicate C-fibre. Scale bars = 200 nm. (**a**–**d**) Unilateral row of right root microtubules in ‘U-shape’ near their origin. Singlet root originates in front row of right root microtubules. Left root microtubules are underlain by C-fibre near their point of origination. (**e–i**). Singlet root microtubule runs posteriorly, joining row of left root microtubules. Right root splits into two parts. Inner part (2 microtubules) runs adjacent to assembly of singlet root and left root. Outer right root microtubules spread out and support right rim of groove. (**j**–**m**) Left root splits into two parts. Inner part of left root (MTs 1–3) splits again into two parts. Inner part of left root (MTs 1–2) runs together with singlet root, and in parallel with inner right root. MT 3 runs separately. Outer part (MTs 4–7) runs posteriorly, supporting left rim of groove.

**Figure 4:**
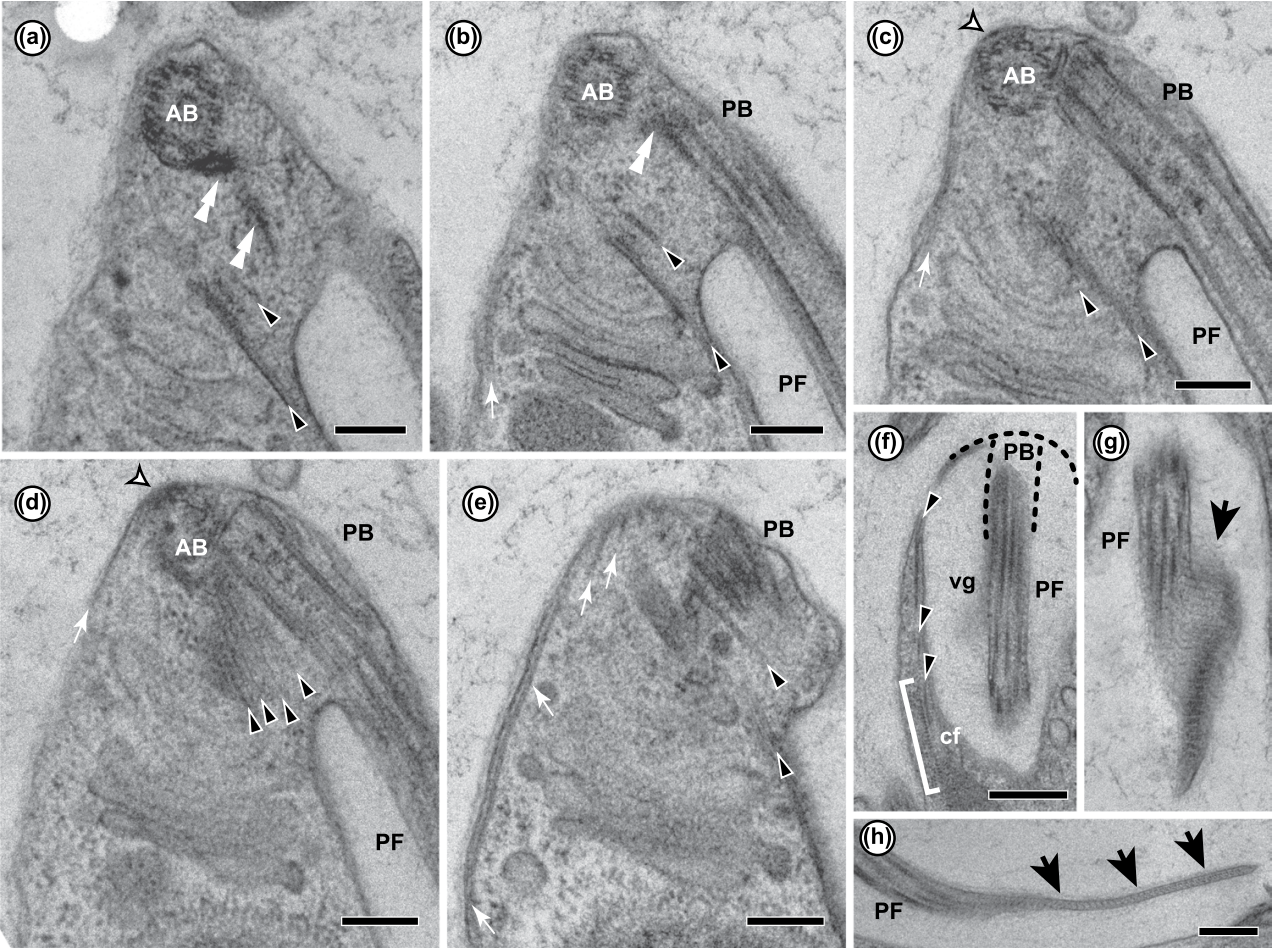
Transmission electron micrographs of *Ophirina amphinema*. AB, anterior basal body; PB, posterior basal body; PF, posterior flagellum; vg, ventral feeding groove; cf, composite fibre. Black arrowheads and arrows indicate right root microtubules and flagellar vane, respectively. White arrowheads and arrows indicate organizing centre for dorsal fan microtubules and dorsal fan microtubules, respectively. White double arrowheads indicate B-fibre. (**a**–**e**) Five consecutive serial sections showing origin of dorsal fan microtubules and apparent lack of distal fibres connecting basal bodies. Note that (c) is higher-magnification view of same cell and section shown in Fig. 2a. Scale bars = 200 nm. (**f**) Right root microtubules supporting right rim of groove, accompanied by composite fibre showing thin banded patterns. Position of posterior basal body and anterior outline of cell projected from nearby sections. Scale bar = 500 nm. (**g**) Thin fibrous structures supporting flagellar vane from inside are spaced at regular intervals and exist at right angle to flagellar axis. Scale bar = 200 nm. (**h**) Lateral section of flagellar vane shows tubular supporting structure. Scale bar = 400 nm.

The flagella were naked and devoid of scales or hairs; however, the posterior flagellum bore two vanes (Figs 1e,f, 2j–l). The anterior flagellum tended to curve semi-posteriorly, while the posterior flagellum extended alongside or through the ventral groove (Fig. 1e,f). On the posterior flagellum, one vane, oriented ventrally (i.e., away from the centre of the cell) and to the right, originated almost at the point of flagellar insertion (Figs 2j, 3f–j). The other vane, oriented on the opposite side of the flagellum (i.e., dorsal — projecting into the groove — and to the left), did not appear until midway down the cell (Figs 2k,l, 3k–m). Each vane was supported internally by thin striated material (Fig. 4g,h) extending from, but not visibly connected to, the axoneme (Figs 2j–l, 3f–m). No other accessory structures, either associated with the vane or not, were observed.

The flagella arose from two basal bodies at the extreme anterior apex of the cell, arranged at a right angle to one another (Fig. 2a). Each basal body had a longitudinally-segmented core structure, extending from within the triplet region almost to the transition zone (Fig. 4c,d). The two basal bodies were connected by the ‘B-fibre’ (see below) from the ventral side (Fig. 4a,b), and by a small ‘pad’ of material immediately abutting the proximal end of the posterior basal body (Fig. 4c,d).

The ventral groove was supported by three microtubular roots, all of which extended from the base of the posterior basal body (Figs 2a,f–l, 3a–m). One of these, the ‘right root’, was composed of ca. 20 microtubules; near the posterior basal body, it was sandwiched between a layered and striated ‘A-fibre’ on the cytoplasmic (dorsal) side and a thick, complex ‘I-fibre’ on the membrane (ventral) side (Fig. 2m). The I-fibre consisted of a thin (~25 nm) electron-dense layer, possibly comprising two appressed sheets, on the ventral side, and a thicker (50–100 nm) electron-lucent structure with faint regular internal differentiation, and was associated with thin fibrous structures extending from the main sheet-like structure of the right root microtubules (Fig. 2m). Although the A-fibre was closely appressed to about the outer (rightmost) ~half of the microtubules of the right root, it separated from the right root as it approached the basal body, with its tip connecting to the posterior basal body two doublets removed from the nearest right-root microtubule (Fig. 2m). The outer (rightmost) and ventral side of the I-fibre connected to the electron-dense and internally-unstructured ‘B-fibre’. The B-fibre arced ventrally, passing close by the posterior basal body to extend anteriorly, and appeared to connect to the anterior basal body (Figs 2e–g, 3a–e). Near the posterior basal body, the right root showed a broad “U” shape (Fig. 2m), with the convex surface dorsal, adjacent to the Golgi apparatus. The right root split into two parts, just distal to the location of the axosome on the posterior basal body, and just proximal to the point of flagellar emergence. The ‘inner’ part, consisting of two microtubules, extended straight and posteriorly, while the ‘outer’ part curved rightward to the margin of the feeding groove. The inner microtubules of the outer part frayed apart (Figs 2g–i, 3e–m). Near the posterior end of the cell, the right rim of the groove was supported by the outer part of the right root and an associated compound nonmicrotubular structure, the ‘composite fibre’ (Figs 2k–l, 4f).

The left portion of the groove was underlain by the two other posterior roots. The ‘left root’ was composed of seven microtubules, numbered 1–7 from the basal body outward. The associated ‘C-fibre’ was an electron-dense fibrous structure on the dorsal side of the left root. It attached to the posterior basal body and curved leftward to the outside (left) edge of the left root (Fig. 3b–g), and was not directly connected to the left root microtubules (Fig. 3d–f). The third root, a ‘singlet root’ consisting of a single microtubule, originated in the space between the right root microtubules, the posterior basal body, and the A-fibre (Figs 2m, 3b). This ran posteriorly and leftward, joining the left root (Fig. 3b–m). No fibres were observed specifically associating with this root (Fig. 3b–m). The left root divided into two parts ~450 nm distal to its point of origin. The ‘inner left root’ comprised microtubules 1–3, which, along with the singlet root, followed a relatively straight trajectory, close to the midline of the groove. The ‘outer left root’ comprised microtubules 4–7, and followed the left margin of the groove (Fig. 3j–m). Within 140 nm posterior to this, the inner left root divided again, with microtubules 1–2 and the singlet root running posteriorly in parallel with, and not far from, the inner right root. Left root microtubule 3 ran separately to the posterior of the cell, between the outer and the remainder of the inner left root. The complex formed by the singlet and the inner left root, and separately, microtubule 3 of the left root, supported the middle-left portion of the ventral groove (Fig. 3k–m). The outer left root microtubules continued to support the left rim of the groove for as far posteriorly as we were able to trace them (Figs 2l, 3j–m).

The anterior basal body was associated indirectly with several microtubules, which originated at an electron-dense structure positioned between them and the basal body (Fig. 4c,d). These microtubules formed a dorsal fan, spreading apart as they extended rightward and posteriorly along the dorsal side of the cell. No other cytoskeletal microtubules, such as would be regarded as anterior roots, could be recognized (Fig. 4a–e).

**Figure 5:**
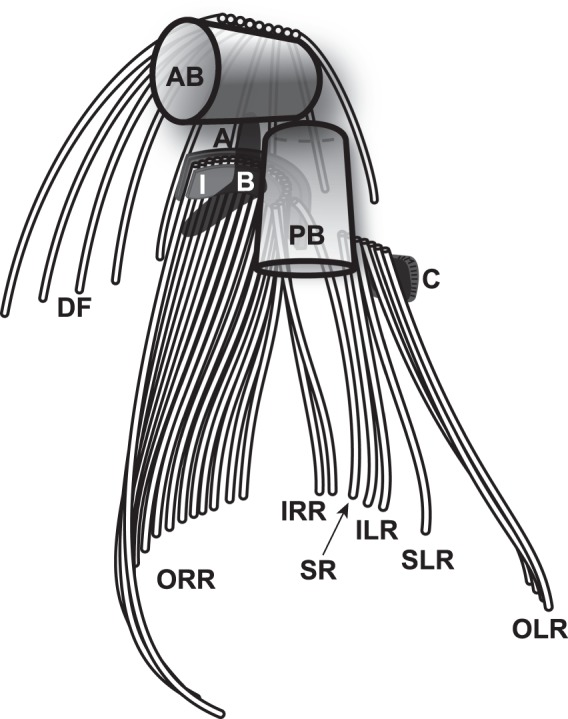
Diagrammatic representation of proximal flagellar apparatus of *Ophirina amphinema* viewed from ventral face, showing basal bodies, microtubular flagellar roots, and non-microtubular components. AB, anterior basal body; PB, posterior basal body; A, A-fibre; B, B-fibre; C, C-fibre; I, I-fibre; ORR, outer right root microtubules; IRR, inner right root microtubules; SR, singlet root microtubule; ILR, inner left root microtubules; SLR, singlet left root microtubule; OLR, outer left root microtubules; DF, dorsal fan microtubules.

A reconstruction of the cytoskeletal structure, as based on these findings, is shown in Fig. 5.

### Phylogeny

**Figure 6:**
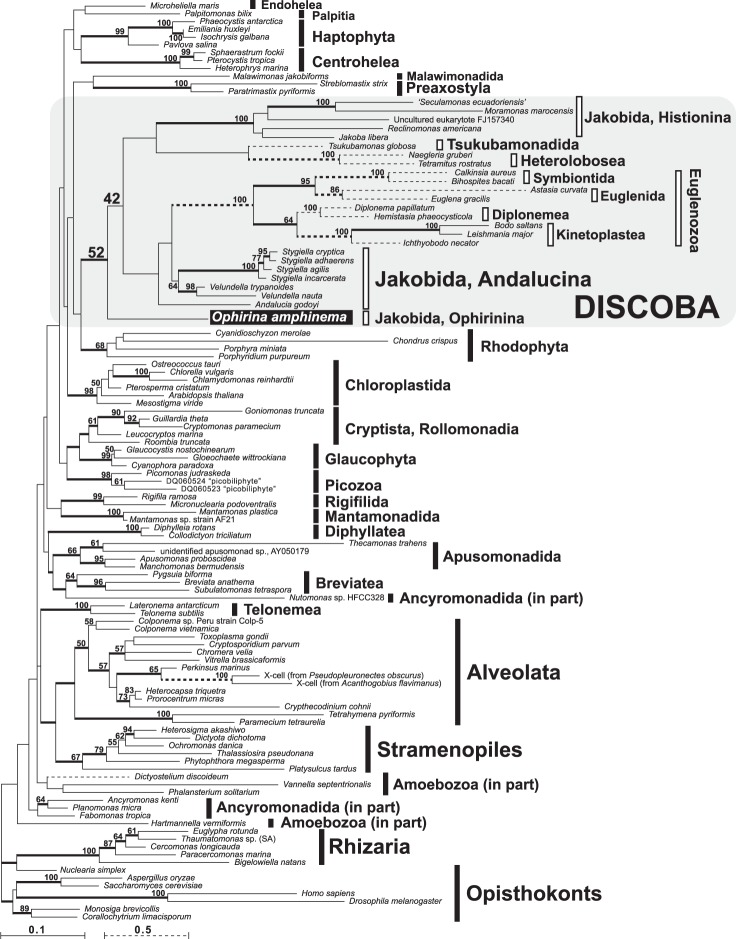
Phylogenetic position of *Ophirina amphinema* inferred from maximum-likelihood (ML) analysis of 18S rDNA including 1,362 nucleotide positions. Bootstrap values ≥ 50% are labelled. Nodes supported by Bayesian posterior probabilities ≥ 0.95 are shown by thick lines.

In our 18S rDNA tree, *Ophirina amphinema* was sister to Discoba; this topology, however, was only weakly supported (Fig. 6). Discobid assemblages, as well as relationships within Discoba, were at best poorly resolved, with the exception of Euglenozoa, whose monophyly was recovered with maximum statistical support. For instance, jakobids did not form a clade, even when *O*. *amphinema* was excluded from the dataset (data not shown). The monophyly of other well-supported eukaryotic groups, though, such as Chloroplastida, Rhizaria, and Haptophyta, was recovered in our analysis, consistent with previous results by others (e.g^14,15^).

**Figure 7:**
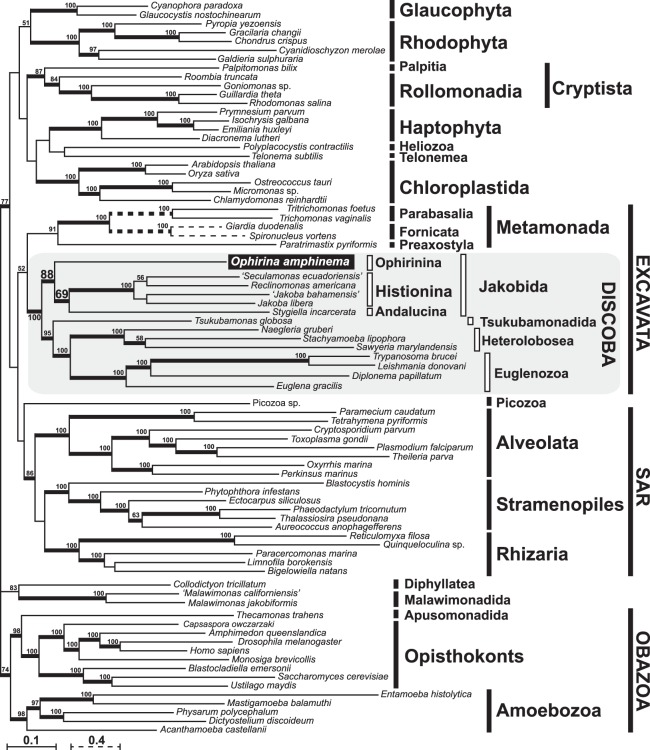
Phylogenetic position of *Ophirina amphinema* inferred from maximum-likelihood (ML) analysis of 156-gene alignment comprising 40,903 amino acid positions. Bootstrap values ≥50% are labelled. Nodes supported by Bayesian posterior probabilities ≥0.95 are shown by thick lines.

Trees from our 156-protein analyses (Fig. 7) are generally consistent with those shown in Yabuki *et al*. (2014^16^), upon which our present dataset was based. The monophyly of major eukaryotic assemblages, such as Cryptista, SAR, and Obazoa, was recovered with moderate to strong bootstrap support. Further, the phylogenetic affiliation of some protist lineages that were not well-placed in the 18S rDNA tree, such as that of *Palpitomonas* and *Tsukubamonas*, was well resolved. The monophyly of Discoba (including *O*. *amphinema*) was recovered with maximal support. The internal relationships among the four subgroups of Discoba (i.e., Jakobida, Tsukubamonadida, Heterolobosea, and Euglenozoa) were also clearly resolved with high to maximal statistical support, with a basal split between jakobids + *O*. *amphinema* on one hand, and the remaining three lineages on the other, and with *Tsukubamonas* sister to Discicristata (Heterolobosea + Euglenozoa, recovered with maximal support). Jakobida, as represented by previously-sequenced members, was recovered but not robustly supported, but *O*. *amphinema* and previously-sequenced jakobids formed a clade with good support. While *O*. *amphinema* and Andalucina were each represented by only a single exemplar, Histionina was represented by four, and these formed a clade that received maximal support.

### Mitochondrial genome architecture

**Figure 8:**
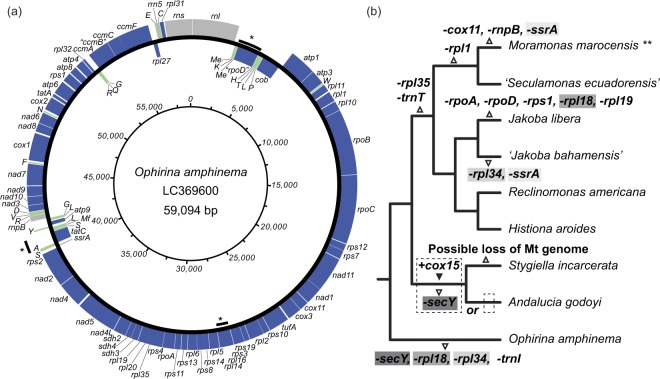
Mitochondrial genome architecture of *Ophirina amphinema* and evolutionary changes in jakobid mitochondrial genomes. (**a**) Circular-mapping genome. Protein-coding regions are shown in blue. Transfer RNA genes and other structural RNA genes (i.e., *rns*, *rnl*, *Rrn5*, *rnpB* and *ssrA*) are shown in green and grey, respectively. Asterisks indicate regions in which genes overlap (see Supplemental Information Fig. S1). (**b**) Putative gene loss and gain events during mitochondrial genome evolution in jakobids. Parallel losses of same genes are shown with correspondingly shaded grey backgrounds. **Complete sequence of mitochondrial genome of *Moramonas marocensis* is not yet released, therefore reductive evolution of genome on branch leading to *M*. *macrocensis* is incompletely represented.

The circular-mapping mitochondrial genome of *O*. *amphinema* is 59,094 bp in length (Fig. 8a). A total of 94 genes were annotated on this genome, comprising three ribosomal RNAs (*rnl*, *rns* and *rrn5*), one RNase-P RNA (*rnpB*), one transfer-messenger RNA (*ssrA*), 63 protein-coding genes, and 26 tRNA species. Two genes were initially identified as ORFs but without functional annotation by MFannot; we annotated them as *rpoD* and *ccmB* based on the results of DELTA-BLAST. Introns were not detected in any ORFs. Three *trnM* (cau) sequences were detected: BLASTN analysis suggested that two of them were of the elongation type, and that the remaining one was of the initiator type. The sequences of the two elongation-type *trnM* (cau) genes were different to each other.

Of the 59,094 bp in the mitochondrial genome, estimated coding regions accounted for 55,603 bp (94.1%). The calculated average sequence length of intergenic space was 37 bp. Furthermore, we detected three regions in which two genes overlapped, one of which overlapped over 60 bp (Fig. 8a and Supp. Fig. 1). Possible Shine-Dalgarno motifs (5′-AAG-3′) were also detected at upstream flanking regions of more than half of the genes (Supp. Fig. 2). The annotated mitochondrial genome sequence was deposited in GenBank with accession code LC369600.

## Discussion

In this study, we isolated and characterized a new deeply-branching member of “Excavata”, *Ophirina amphinema* gen. et sp. nov., using a combination of morphological and molecular sequence data. Light-microscopic observation clearly revealed the existence of a ventral feeding groove in *O*. *amphinema* (Fig. 1a,b), which is a hallmark characteristic of (though not exclusive to) Excavata^17^. Although cell dimorphism has not frequently been reported from other excavates, a suspension-feeding cell with a groove and a swimming cell with a longer anterior flagellum were reported from the jakobid *Stygiella incarcereta*^18,19^. Our phylogeny based on the 18S rRNA gene showed a sister relationship between *O*. *amphinema* and the entirety of discobids, but without significant bootstrap support (Fig. 6). Without further data, these observations suggest that this new flagellate represents a new deep branch within the eukaryotes.

Phylogenetic analyses based on the use of our 156-protein dataset are generally consistent with what was shown in Yabuki *et al*. (2014^16^), indicating that the addition of the *O*. *amphinema* data did not disrupt the relationships of other taxa. Likewise, the addition of this novel deep-branching taxon did not resolve the lack of unity of excavate taxa: Discoba branched without significant support as sister to Metamonada, and separately from Malawimonadida. The 156-protein tree showed that *O*. *amphinema* branches within Discoba with the highest statistical support, which is consistent with (although not statistically supported by) 18S rDNA phylogeny. Further, *O*. *amphinema* is shown either to be closely related to, or to belong within, Jakobida. Based on phylogenetic and ultrastructural data (see below), we thus consider *O*. *amphinema* to be a member of Jakobida, but without specific affiliation to neither of the two currently-described suborders of jakobids. Instead, we place the flagellate in the new jakobid suborder Ophirinina.

From the perspective of ultrastructure, *O*. *amphinema* possesses characteristic subsets of the ‘typical excavate’ flagellar apparatus. In many regards, *O*. *amphinema* presents a mosaic of characteristics seen in different excavate subgroups. Note that our comparative analyses as summarized in Table 1 include representatives from all three main sub-lineages of the “supergroup” Excavata despite the observations that these sub-lineages rarely all group together in phylogenetic analyses. This recognition nonetheless persists because many members of each of these three lineages share a strikingly similar ‘typical excavate’ cell morphology, a suite of features not found together in other eukaryotes. This morphology is largely based around a longitudinal suspension-feeding groove. A posteriorly-directed flagellum runs through the groove and, assisted by one or more membrane-bounded vanes with a striated internal support, creates a feeding current that carries food particles through the groove. Internally, the groove is supported by three predominantly-ribbon-like microtubular ‘roots’ attached to the posterior flagellum’s basal body; the root on the right side of the groove splits into two parts (e.g.^4,17,20^), and the central root is almost always a single microtubule. Various non-microtubular fibres, with substructures consistent across all three groups of excavates, and which are not known outside of excavates, are associated with these roots^17^. Some other aspects of this morphology nevertheless have been found in other eukaryote lineages as well^21^, which is expected if a subset of ‘typical excavate’ morphology indeed represents characteristic of the last eukaryotic common ancestor^21,22^.

**Table 1:** Comparison of ultrastructural features of *Ophirina amphinema* gen. et sp. nov. and other ‘typical excavates’.

Taxon	Number of flagellar vanes	AR	A fibre	Origin of B fibre	CF	DF	mitochondrial cristae	SA	Distal fibre between AB and PB
*Ophirina*	2	−	+	PB/AB	+	+	F-D	−	−
Jakobids (Histionina)	1 (D)	−	+	PB	+	+	F/T	+/?	+/−
Jakobids (Andalucina)	1 (D)	*	+	PB	+	+	T/−	+	+
*Tsukubamonas globosa*	0	+	+	PB	−	−	T	+	−
*Trimastix*	2	+	+	RR	+	+	−	+	−
*Carpediemonas* & related organisms (CLOs)	2–3	+/−	+/−	LR	+/−	+/−	−	+/−	+/−
Retortamonads	2–3	+/−	+/?	LR	+	+/−	−	?	−
*Malawimonas*	1 (V)–2	+	+	PB	+	+	D	?	+

+, present; −, absent; ?, uncertain; AR, anterior root; CF, composite fibre; DF, dorsal fan; SA singlet root associated fibre; PB, posterior basal body; AB, anterior basal body; LR, left root; RR, right root; F, flat cristae; T, tubular cristae; D, discoid cristae; (D), dorsal vane only; (V), ventral vane only. Presently-identified AR of jakobids (*) is probably not homologous to AR of other excavates.

The flagellar vane is one of the features suggesting that *O*. *amphinema* is a deep-branching member of the discobids. The posterior flagella of all jakobids examined so far possess only one (dorsal, i.e., groove-facing) flagellar vane^14,19,23–26^, whereas *O*. *amphinema* has two, which is probably the ancestral state for metamonads^27^ and malawimonads^22^ (see also discussion below). The left/right displacement of the vanes, however (perhaps best visualised as a clockwise torsion of the vane system when viewed base-to-tip), is not known in other excavates, at least not to the degree seen in *O*. *amphinema*, and may represent a unique character. Likewise, the segmented core structure of the basal bodies is unknown in other excavates. On the other hand, while the B-fibre connects the right root to the left root in the fornicates (one of the main groups within Metamonada), the B-fibre of *O*. *amphinema* instead connects the right root to the posterior basal body, and continues from there to connect to the anterior basal body. Such an arrangement of the B-fibre is rather more similar to that of the jakobids than it is to that of the other excavates^23,28^. In addition, the dorsal fans of both jakobids and *O*. *amphinema* originate from non-microtubular MTOCs appressed to their anterior basal bodies. These dorsal fans generally run antiparallel to their associated basal bodies (i.e., their microtubules run in directions close to the opposite of those in the associated axoneme). The sole known exception to this is *Stygiella*, in which the fan is directed leftward from the anterior basal body; however, in this organism the anterior basal body itself is rotated to the left, such that the anterior flagellum projects leftward from the cell. With regard to the cell itself, then, the dorsal fan in *Stygiella* has the expected orientation, as it is directed primarily dorsally and posteriorly without significant bias to either left or right, and it is only the anterior basal body’s orientation that differs from other jakobids. In contrast, *O*. *amphinema*’s anterior flagellum projects primarily medially (as is the case for most excavates), and its fan is directed to the right of the cell. Other excavates, including both metamonads and malawimonads, have an anterior root that arises from the anterior surface of the anterior basal body. This root heads to the left of the cell before it turns posteriorly, and the dorsal fan originates from that anterior root^17^. In these cases, while the fan is thus proximately associated with a leftward-running root, it is ultimately oriented antiparallel to the anterior basal body, just as in jakobids. Amongst all known excavates, then, the rightward-oriented fan of *O*. *amphinema* is unique. The absence of a singlet-root-associated fibre also distinguishes *O*. *amphinema* from many other excavates, including other jakobids. A summarised comparison of the ultrastructural characteristics of *O*. *amphinema* with those of other excavates is presented in Table 1, in which it can be seen that *O*. *amphinema* has a unique combination of ultrastructural characters as a whole.

We may note that the presence of two vanes on *O*. *amphinema*’s posterior flagellum eliminates one of the characters formerly thought of as distinguishing discobid ‘typical excavates’: as mentioned above, all other jakobids have only a single dorsal vane. This similarity extends beyond the mere number of vanes on the flagellum: the ventral vane arises very close to the point of flagellar emergence, and the dorsal considerably more distally, in *O*. *amphinema*, metamonads (e.g.^22,29^). and malawimonads^30^. This, along with the perpendicular striations of the material within the vanes, suggests strongly against the vanes being a convergent character. Vanes are known in other eukaryote groups as well, such as some alveolates^31^ and stramenopiles^32^, but in these groups the vanes lack the obvious striated electron-dense material found in excavates, and perhaps as a result appear less rigid. Vanes are also known (or suspected to be found) in most if not all choanoflagellates (e.g.^33,34^), but in this case, these vanes have a markedly different structure, being much wider, possibly thinner, and apparently composed of fibrillar material, and are unlikely to be homologous to those of excavates. Regardless, the vanes of malawimonads, metamonads, and discobids (including *O*. *amphinema*) resemble one another far more than they do any other comparable (non-excavate) structures.

This newfound morphological unity, both in terms of microtubular and nonmicrotubular intracellular morphology and in terms of flagellar vanes, stands in contrast both to our own phylogenies and to recent studies^30,35^ that have cast increasing doubt on ever establishing the monophyly of the three subgroups of excavates. ‘Typical excavate’ morphology may well be ancestral to most or all extant lineages of eukaryotes^30^, and those characters that *O*. *amphinema* bears in common with non-discobid excavates underscore just how precisely we may draw a portrait of a eukaryotic cell that lived over a billion years ago.

The discovery of *O*. *amphinema* also contributes to a more precise understanding of mitochondrial genome evolution within jakobids. The mitochondrial genome of *O*. *amphinema* is generally similar to those of the other jakobids in respect to gene-richness. Several genes identified in the mitochondrial genome of *O*. *amphinema* (e.g., *rpo A–D*) are otherwise found in the mitochondrial genomes of jakobids only. The high gene density is also a shared characteristic with the other jakobids (outside of *Moramonas*,^26^). The proportion of the coding region in the mitochondrial genome in *O*. *amphinema* (94.1%) is higher than that of the other known jakobids, although that of *Chondrus crispus* (Rhodophyta) is higher (96%,^36^). The mitochondrion of *Andalucia godoyi* has been considered the closest modern example to that of the ancestral jakobid; it possesses the most slowly evolving mitochondrial genome in terms of gene content^7^. The 156-protein tree suggests that *O*. *amphinema* is also an early-branching jakobid; and, as one would expect, its mitochondrial genome is gene-rich, although it encodes seven fewer genes than that of *A*. *godoyi*. This supports the notion that parallel reduction of the mitochondrial genome occurred within the jakobids. For example, *secY* was likely lost in parallel along the branches leading to *O*. *amphinema* and to *A*. *godoyi*. Likewise, *rpl18* was lost on two separate occasions after the divergence of *O*. *amphinema* and *Jakoba libera* (Fig. 7b). As for the number of such losses, we note that two of the genes lost from *O*. *amphinema*’s mitochondrial genome (*rpl18* and *secY*), are syntenic in all other jakobid lineages having both genes, and therefore may have been lost from *O*. *amphinema* in a single gene-loss event. This implies that the reduction is less significant than the raw number of lost genes might suggest. Since both mitochondrial genome architecture and mitochondrial gene complements are diverse in eukaryotes, and independent parallel losses of mitochondrial genes have been inferred from various eukaryotic lineages (e.g.^5,9^), it is not surprising that such parallel reductive evolution may have also occurred within jakobids. Our findings indicate afresh the plasticity of the mitochondrial genome.

### Taxonomy

Excavata, Discoba, Jakobida

Ophirinina n. subord. Yabuki, Gyaltshen, Heiss, and Kim

**Description:** Unicellular heterotrophic biflagellated protists with ventral suspension-feeding groove. Basal bodies with segmented electron-dense core material. Posterior flagellum possesses two vanes, oriented dextroventrally and sinistrodorsally, former developing at point of flagellar emergence and latter somewhat distally to that. Groove supported by left and right posterior microtubular roots, with intermediate singlet root, and A-, B-, C-, and I-fibres. Anterior roots absent; dorsal fan present, organised by non-microtubular MTOC adjacent and immediately anterior to anterior basal body. B-fibre connects both basal bodies. Singlet-root-associated (SA) fibre absent. Fan projects posteriorly and rightward within cell. Mitochondrion with flat to discoid cristae.

Ophirinidae n. fam. Yabuki, Gyaltshen, Heiss, and Kim (ICZN)

**Description:** Same as suborder

**Type genus:** *Ophirina*

*Ophirina* n. gen. Yabuki, Gyaltshen, Heiss, and Kim (ICZN)

**Description:** Same as family.

**Type species:** *Ophirina amphinema*

**Etymology:** *Ophirina* is derived from Ophir, which is the name of a faraway land famously known for its wealth. It is the place from which the Queen of Sheba was said to have brought gold and other treasures to King Solomon. While the exact location of Ophir has always been uncertain, there are several geographical places that have been linked to this legendary land. One such proposed locale is the Solomon Islands, which group was named after King Solomon by Alvaro de Mendana de Neira, a European sailor who declared, upon his arrival in the islands, to have found Ophir. While the search for the source of Solomon’s gold is actually still ongoing in the nation, it is clear at least that this South Pacific country holds great wealth in the form of microbes^37^, including the flagellate described in this study.

*Ophirina amphinema* n. sp. Yabuki, Gyaltshen, Heiss, and Kim (ICZN)

**Description:** Cell 3.9 ± 0.4 µm in length. Two types of cell morphology present, both flagellated, with one type a largely stationary suspension feeder and the second a nodding swimmer. Both flagella as long as cell length in suspension feeder. Both flagella ca. twice as long as cell length in swimmer.

**Hapantotype:** SEM stub (IZC 00267134) deposited in the American Museum of Natural History. This specimen, an hapantotype, is the name-bearing type for the species. Figure 1f depicts an example from this specimen.

**Type locality:** Nusa Lavata lagoon, Solomon Islands (8°46′29.92′′S, 157°46′20.20′′E). Note that this location was referred to as “Nirasa lagoon” (the letter ‘g’ should have been placed following the ‘N’) in an earlier study that found a surprisingly high level of novel diversity of deeply branching eukaryotes by metabarcoding approach^37^. While the island Ngirasa is located in the vicinity of the collection site, it recently came to our attention (thanks to Dr. Simon Albert) that the island immediately bordering the collection site is called Nusa Lavata.

**Etymology:** The specific epithet *amphinema* (amphi meaning “double”; nema meaning “filament”) refers to the organism’s dimorphism, which prominently includes difference in flagellar length between morphotypes.

**Gene sequence:** 18S rRNA gene sequence (GenBank accession LC369601)

**Distribution and habitat:** Known from Nusa Lavata (previously published as “Nirasa”) lagoon, an oligotrophic tropical lagoon.

**Zoobank registration:** urn:lsid:zoobank.org:pub:C03FC0A1-D290-4B6F-A393-B0F6D90DEFA6

## Methods

### Strain establishment and cell culturing

A water sample from Nusa Lavata lagoon (formerly referred to as “Nirasa” in previous accounts, e.g.^37^) of the Solomon Islands was inoculated and maintained as an initial enriched culture at 23 °C in Erd-Schreiber medium (ESM) as specified by the Japanese National Institute for Environmental Studies (http://mcc.nies.go.jp/02medium.html). The enrichment culture was then serially diluted across a 96 well plate, which was followed by manual single-cell isolation using a hand-pulled micro-pipette. The established culture (strain JB) was then maintained in varying conditions, including (i) ESM fortified with 2.5% Cerophyl medium (ATCC 802), (ii) a 1:1 mixture of ESM and L1, the latter as specified by the National Center for Marine Algae and Microbiota (https://ncma.bigelow.org/algal-recipes), each at 23 °C, and (iii) in KLB medium^38^ at 20 °C. The cells in each growth condition grew well, and showed no apparent morphological difference.

### Light microscopy

Living cells were observed under an Axiovert 100 microscope (Zeiss) using differential interference contrast (Nomarski) optics. Images were obtained with an Olympus DP73 digital camera.

### Scanning electron microscopy

Two different fixation approaches were applied to prepare the specimens for SEM. In each, concentrated cells were applied directly in medium to a 12-mm circular cover slip coated with poly-L-lysine, and glutaraldehyde was added to a final concentration of 2.5% (v/v). In one, the ‘fast’ preparation, fixation occurred over 60 min; the cells were then washed in two changes of artificial seawater (ASW), postfixed in 1% (w/v) OsO_4_ in ASW for 30 min, rinsed in ASW, 50% ASW, and distilled water (1 min each wash), and dehydrated rapidly in an ethanol series (50–70–85–95–100 × 2, 5 min each change). In the other, the ‘slow’ preparation, glutaraldehyde fixation occurred over 40 min, after which the cells were washed in ASW, then 50% ASW, then distilled water (10 min each wash); postfixed in 2% (w/v) unbuffered OsO_4_ for 30 min, washed again 3x in distilled water (10 min each wash), and dehydrated slowly in an ethanol series (10–30–50–70–90–100 × 3, 10 min each change). Each preparation was dried in CO_2_ at critical point, and finally sputter-coated with Au/Pd. Specimens were observed in a Hitachi S-4700 SEM, with an accelerating voltage of 5 keV and an emission current of 15 µA, at a working distance of ~11 mm, using a secondary-electron detector.

### Transmission electron microscopy

A cell pellet was harvested from approx. 300 ml of two-week-old culture by centrifuging at 1,200 x *g* for 4 min, and then was high-pressure frozen (at -196 °C under 2,181 bar) using a Leica EM PACT2 (Leica). The frozen pellet was transferred to 1% osmium tetroxide in HPLC grade acetone and substituted for 118 hours at -85 °C, then warmed to -20 °C over five hours. After this, the specimen was warmed to 4 °C over 48 hours and then washed in acetone. Subsequently, the specimen was transferred through increasing concentrations of Quetol 651 resin (Nisshin EM Co., Ltd.) in acetone: 1:2 resin/acetone for 1 hr, 1:1 for 2 hr, and 2:1 for 3 hr. The specimen was then embedded in pure Quetol 651 resin overnight at room temperature, and this step repeated again with fresh resin. The embedded specimen was polymerized overnight in fresh resin at 65 °C.

Ultrathin sections (ca. 70 nm) of both EM blocks were prepared using an ultramicrotome (Ultracut S; Leica), double stained with 2.0% uranyl acetate and 2.0% lead citrate (Sigma-Aldrich Co.), and observed in a Tecnai G2 20 electron microscope (FEI).

### Extraction of DNA and amplification of 18S rDNA

About 300 ml of two-week-old culture were centrifuged at 1,500 x *g* for 5 min. Total DNA was extracted from the resulting pellet using the DNeasy Plant Mini Kit (Qiagen), according to the procedure provided by the manufacturer. The 18S rDNA was amplified with the primer set ‘Euk1A’^39^ and ‘EukB’^40^. Total PCR reaction volume was 10 µl, and amplification consisted of 35 cycles of denaturing at 94 °C for 30 s, annealing at 55 °C for 30 s, and extension at 72 °C for 1 min. The amplified products were gel-purified and then cloned using the StrataClone PCR Cloning Kit (Agilent Technologies), and clones were screened and sequenced. The obtained sequence was added to the alignment used in Yabuki *et al*. (2013^41^), manually aligned in Mesquite 3.10^42^, and then masked to omit ambiguously-aligned positions. The resulting dataset consisted of 1,362 positions and 113 taxa. A maximum-likelihood tree was obtained from this dataset using RAxML v7.2.8^43^ with the GTR + Γ + I model, the topology used being the best of 30 runs. 100 bootstrap replicates were analysed using the same model. A Bayesian analysis was run using MrBayes v3.2.1^44^ with the GTR + Γ + I model. One cold and three heated Markov chain Monte Carlo (MCMC) runs, with default chain temperatures, were run for 1 × 10^6^ generations, sampling trees at 100-generation intervals. 2.5 × 10^5^ generations were discarded as “burn-in”.

### Illumina RNA-seq and 156 gene phylogeny

Approximately 200 ml of mid-exponential-phase culture was subjected to total RNA extraction. Cells were collected onto a 0.8-µm-pore polycarbonate membrane filter under a gentle vacuum condition (>600 mTorr). RNA was extracted from the material adhering to the filter using the TRIzol Plus RNA Purification Kit (Thermo Fisher Scientific), following the manufacturer’s protocol. About 1 µg of total RNA was sent to Cold Spring Harbor Laboratory’s sequencing core, from which a cDNA library was prepared following the TruSeq RNA Sample Preparation v2 protocol, including the poly-A selection step. This library was run on the Illumina NextSeq. 500 system. A total of 123,258,871 paired reads, each up to 150 bp in length, were generated. Raw reads were quality trimmed using Trimmomatic v 0.36^45^ prior to *de novo* assembly using Trinity v 2.3.2^46^. The transcriptome assembly included 58,724 contigs, which were then translated into peptides using TransDecoder v 3.0.1, a built-in module within the Trinity package. A local BLAST database was constructed from the contig sequences, and then 157 gene sequences that were analysed in a previous study^16^ were searched for from it using TBLASTN. The amino acid sequences of the identified genes were added to the alignments analysed in the previous study^16^. Note that α-tubulin was excluded from the dataset in this study because of reported instances of lateral transfer of this gene in the discobids *Andalucia* and *Stygiella*, possibly from a metamonad^47^. Therefore, the final dataset contained 156 protein markers. An ML tree was obtained using RAxML v.7.2.8 under the PROTGAMMALG model (selected as the best fit model by IQTREE^48^) from the best of 10 runs. Bootstrap analysis was based on 100 replicates. A Bayesian analysis was run using PhyloBayes^49^ with the CAT-Poisson model, incorporating among-site rate variation approximated by a discrete gamma distribution. Two Markov chain Monte Carlo (MCMC) runs, each with one ‘cold’ and three ‘hot’ chains, were run for 40,500 generations, sampling log-likelihoods every 250 trees. Bayesian posterior probabilities were calculated after discarding the first 13,250 generations (the point at which the maximum discrepancy between the corresponding bipartition frequencies of the two chains fell <1.00) as ‘burn-in’.

### Sequence and annotation of mitochondrial genome

From the transcriptome assembly, six contigs were identified as possible mitochondrial genomic fragments by BLAST, using the mitochondrial genome sequence of *Andalucia godoyii* as a query sequence. These genome fragments were connected to one another by sequences that were amplified by PCR using heuristic combinations of 12 primers. The primer sequences used in this study are listed in Supplementary Information.

Within the 59,094 bp of circular-mapping mitochondrial genome, open reading frames (ORFs) encoding proteins longer than 50 AA residues were identified by using Artemis^50^ with genetic code 11 (i.e., bacterial and plant plastid), as this code was found in other jakobids^6,7,26^. Each ORF was annotated based on the result of MFannot (http://megasun.bch.umontreal.ca/cgi-bin/mfannot/mfannotInterface.pl), but the position of each stop codon was decided in accordance with the result of the Artemis analysis. The putative amino acid sequences of the three regions that were not annotated by MFannot (i.e., orf119, orf123 and orf217) were subjected to DELTA-BLAST to annotate them based on conserved domain sequences. Infernal v 1.1.2^51^ was also utilized to identify tRNA species using the covariance model analyzed in Nishimura *et al*. (2016^9^). Possible Shine-Dalgarno sequences were identified by manual inspection of the upstream flanking regions of the annotated genes.

## Electronic supplementary material

Supplementary information

Supplementary video 1

Supplementary video 2
