# Supplementary material for: Ophirina amphinema n. gen., n. sp., a New Deeply Branching Discobid with Phylogenetic Affinity to Jakobids
Source: Sci Rep. 2018 Nov 1;8:16219. doi: 10.1038/s41598-018-34504-6 (PMC6212452; doi:10.1038/s41598-018-34504-6)
Supplement: Supplementary file 1 — Supplementary information [file 41598_2018_34504_MOESM1_ESM.pdf]

***Ophirina amphinema* n. gen., n. sp., a New Deeply Branching Discobid with Phylogenetic Affinity to Jakobids**

Akinori Yabuki[1†], Yangtsho Gyaltshen[2], Aaron A. Heiss[2], Katsunori Fujikura[1], Eunsoo Kim[2†]

[1] Department of Marine Biodiversity Research, Japan Agency for Marine-Earth Science and Technology (JAMSTEC)
[2] Division of Invertebrate Zoology and Sackler Institute for Comparative Genomics, American Museum of Natural History

[†]Corresponding authors:
Akinori Yabuki, Department of Marine Biodiversity Research, Japan Agency for Marine-Earth Science and Technology (JAMSTEC)---Telephone number: +81 46867 9498; FAX number: +81 46867-9525; e-mail: yabukia@jamstec.go.jp

Eunsoo Kim, Division of Invertebrate Zoology and Sackler Institute for Comparative Genomics, American Museum of Natural History ---Telephone number: +1 212 769 5510; FAX number: +1 212 769 5277; e-mail: ekim1@amnh.org

Running title: ***Ophirina*, the sister taxon to jakobids**

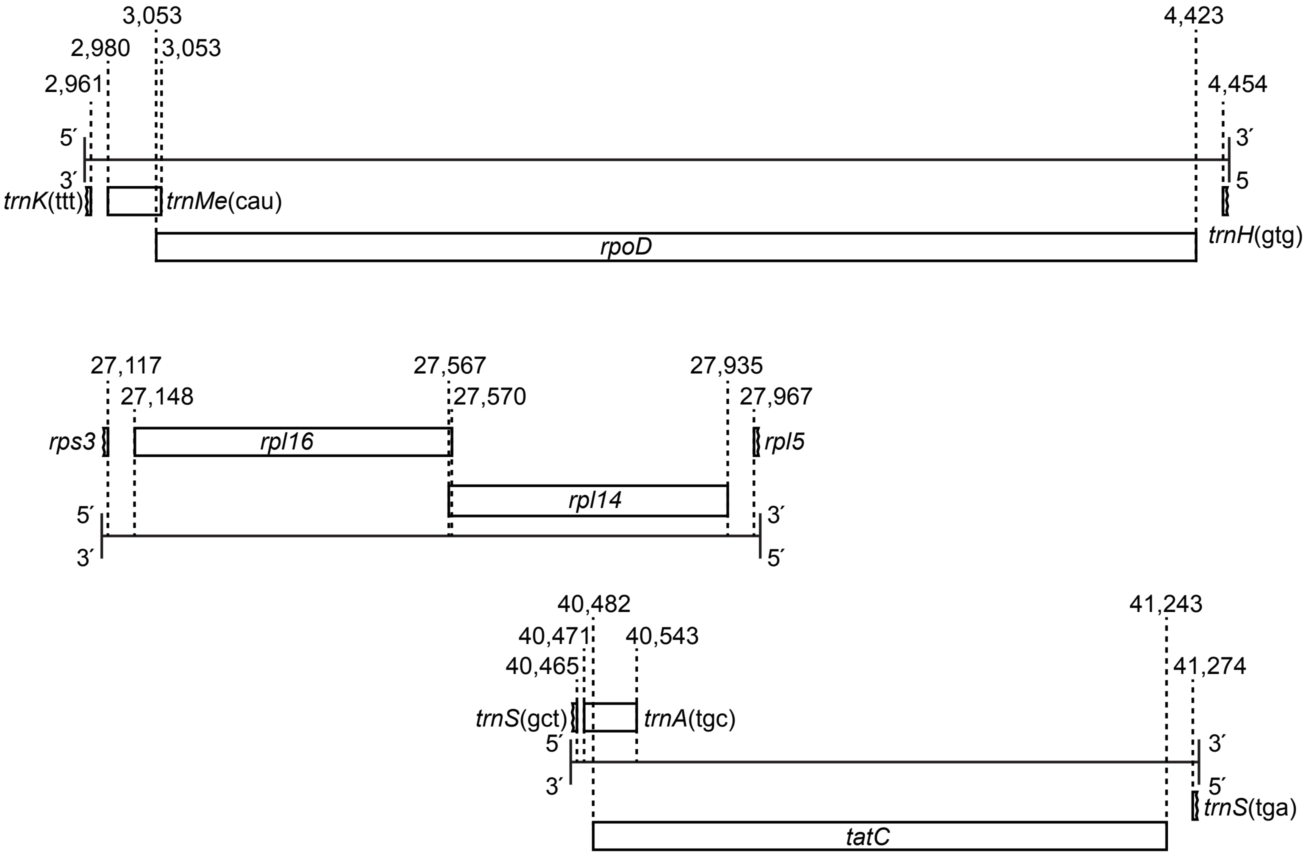
3,053
4,423
2,980
3,053
2,961
4,454
5´
3´
3´
5
trnK(ttt)
trnMe(cau)
trnH(gtg)
rpoD
27,117
27,567
27,935
27,148
27,570
27,967
rps3
rpl16
rpl5
rpl14
5´
3´
3´
5´
40,482
41,243
40,471
40,543
40,465
41,274
trnS(gct)
trnA(tgc)
5´
3´
3´
5´
trnS(tga)
tatC

Fig. S1

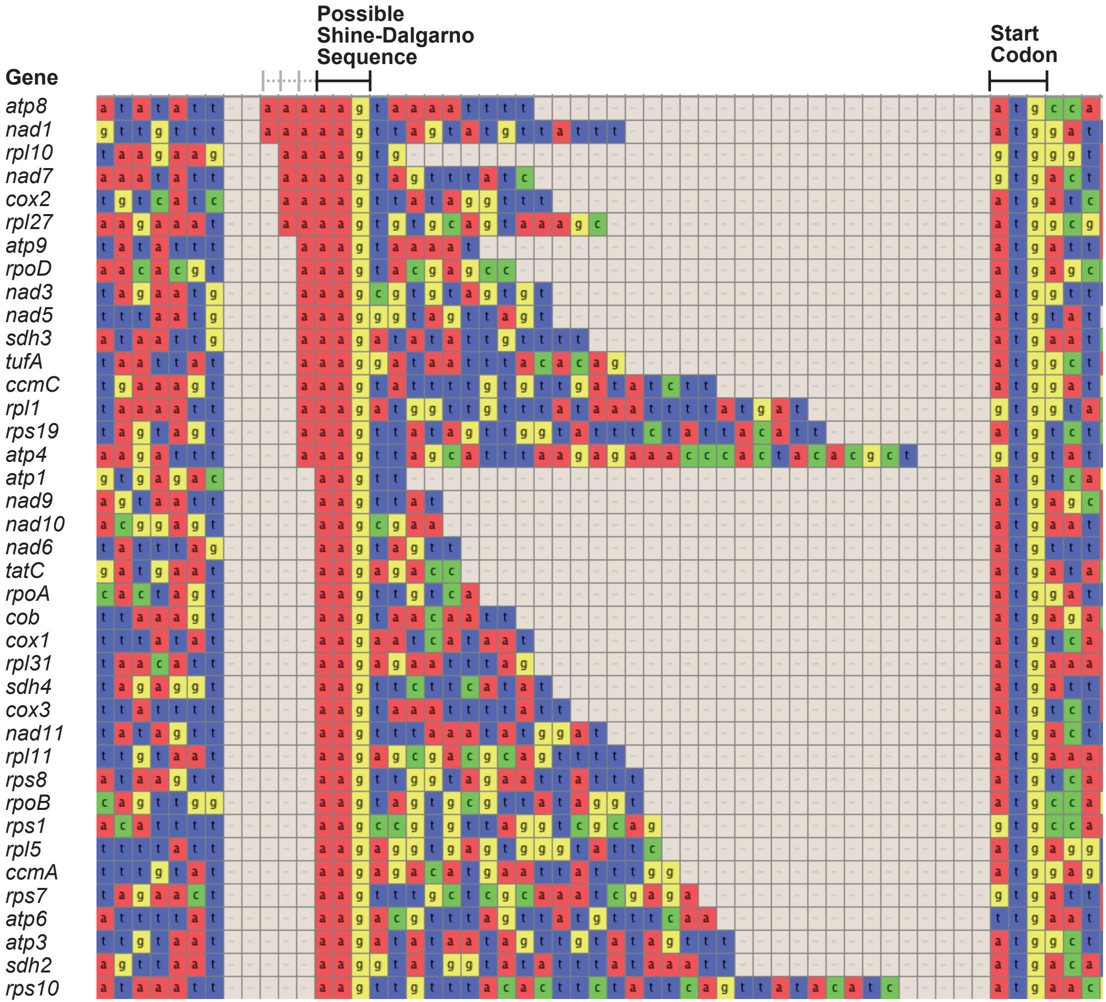
Possible Shine-Dalgarno Sequence
Start Codon
Gene
atp8
nad1
rpl10
nad7
cox2
rpl27
atp9
rpoD
nad3
nad5
sdh3
tufA
ccmC
rpl1
rps19
atp4
atp1
nad9
nad10
nad6
tatC
rpoA
cob
cox1
rpl31
sdh4
cox3
nad11
rpl11
rps8
rpoB
rps1
rpl5
ccmA
rps7
atp6
atp3
sdh2
rps10

Fig. S2

Figure S1. Partial map of the mitochondrial genome in which two genes overlapped.

Figure S2. Possible Shine–Dalgarno translation initiation motifs (5′-AAG-3′).

Supplementary video 1. Two *Ophirina amphinema* cells of suspension type. Note characteristic beating motion of posterior flagellum along feeding groove.

Supplementary video 2. Swimming type cell of *Ophirina amphinema*, showing jittery movement.
